# Transcriptomic risk scores for attention deficit/hyperactivity disorder

**DOI:** 10.1038/s41380-023-02200-1

**Published:** 2023-08-03

**Authors:** Judit Cabana-Domínguez, Natalia Llonga, Lorena Arribas, Silvia Alemany, Laura Vilar-Ribó, Ditte Demontis, Christian Fadeuilhe, Montse Corrales, Vanesa Richarte, Anders D. Børglum, Josep Antoni Ramos-Quiroga, María Soler Artigas, Marta Ribasés

**Affiliations:** 1 Psychiatric Genetics Unit, Group of Psychiatry, Mental Health and Addiction, Vall d’Hebron Research Institute (VHIR), Universitat Autònoma de Barcelona, Barcelona, Spain; 2 Department of Mental Health, Hospital Universitari Vall d’Hebron, Barcelona, Spain; 3 Biomedical Network Research Centre on Mental Health (CIBERSAM), Madrid, Spain; 4 Department of Genetics, Microbiology, and Statistics, Faculty of Biology, Universitat de Barcelona, Barcelona, Spain; 5 Department of Biomedicine/Human Genetics, Aarhus University, Aarhus, Denmark; 6 The Lundbeck Foundation Initiative for Integrative Psychiatric Research, iPSYCH, Aarhus, Denmark; 7 Center for Genomics and Personalized Medicine, Aarhus, Denmark; 8 The Novo Nordisk Foundation Center for Genomic Mechanisms of Disease, Broad Institute of MIT and Harvard, Cambridge, MA USA; 9 Department of Psychiatry and Forensic Medicine, Universitat Autònoma de Barcelona, Barcelona, Spain

**Keywords:** Genetics, ADHD

## Abstract

Attention deficit/hyperactivity disorder (ADHD) is a highly heritable neurodevelopmental disorder. We performed a transcriptome-wide association study (TWAS) using the latest genome-wide association study (GWAS) meta-analysis, in 38,691 individuals with ADHD and 186,843 controls, and 14 gene-expression reference panels across multiple brain tissues and whole blood. Based on TWAS results, we selected subsets of genes and constructed transcriptomic risk scores (TRSs) for the disorder in peripheral blood mononuclear cells of individuals with ADHD and controls. We found evidence of association between ADHD and TRSs constructed using expression profiles from multiple brain areas, with individuals with ADHD carrying a higher burden of TRSs than controls. TRSs were uncorrelated with the polygenic risk score (PRS) for ADHD and, in combination with PRS, improved significantly the proportion of variance explained over the PRS-only model. These results support the complementary predictive potential of genetic and transcriptomic profiles in blood and underscore the potential utility of gene expression for risk prediction and deeper insight in molecular mechanisms underlying ADHD.

## Introduction

Attention deficit hyperactivity disorder (ADHD) is a neurodevelopmental disorder characterized by inappropriate levels of inattentiveness, hyperactivity, or impulsivity that affects around 2.6% of persistent adult ADHD and 6.8% of symptomatic adult ADHD [1]. ADHD increases the risk of health problems, psychiatric co-morbidities, psychological dysfunction, social disability, academic and occupational failure, and risk behaviours throughout the individual’s life [2].

Twin and family studies show a strong genetic component underlying the disorder, with a heritability of 76% [3, 4]. Recently, the largest genome-wide association study meta-analysis (GWAS-MA) on ADHD so far in 38,691 individuals with ADHD and 186,691 controls identified 27 hits for the disorder [5]. In addition, to date more than 40 relevant studies on polygenic risk scores (PRS) for ADHD have been published and show evidence of association between ADHD-PRS and a wide range of traits and disorders, including ADHD-related traits, reduced brain volume, lower education attainment, externalizing behaviours, impaired working memory, higher body mass index or lower socioeconomic status, among others [6].

The SNP-based heritability for ADHD estimated so far is 0.14 [5] and the PRS for the disorder explains 5.5% of phenotypic variance in individuals of European ancestry [7]. A large proportion of the heritability still needs to be explained and gene expression, which results from the interplay between genetic and environmental factors, may help to elucidate additional phenotypic variance. To date, eight studies on transcriptome profiling in ADHD have been performed and highlighted genes involved in several neuronal functions and in the immune system [8–16]. However, this approach is limited by the inaccessibility of brain samples and has mainly focused on blood. Alternatively, integrative approaches have been developed, including transcriptome-wide association studies (TWAS), which are a powerful method to integrate GWAS data and multi-tissue expression quantitative trait loci (eQTL) to correlate genetically predicted gene expression levels with complex traits. To date, four TWAS on ADHD have been performed: three using summary statistics from the first GWAS-MA on ADHD by Demontis et al. [7, 17–19] and one using data from the latest GWAS-MA on ADHD [5]. Briefly, Fahira et al. conducted multiple TWAS approaches to identify 47 putative causal genes and the glutamate receptor signalling pathway underlying ADHD [17]. Liao et al. performed TWAS on 11 brain tissues and identified novel genes and several pathways relevant for ADHD, including the dopaminergic neuron differentiation and norepinephrine neurotransmitter release cycle [18]. Qi et al. considered Chinese and European ancestry cohorts and did not identify transcriptome-wide associated genes with the disorder either in brain or blood [19]. Finally, Demontis et al. identified 23 distinct genes with differential predicted gene expression in the dorsolateral prefrontal cortex (DLPFC) in ADHD using the largest GWAS-MA on ADHD to date and highlighted *PPP1R16A* and *B4GALT2* as top genes [5].

Given that a substantial proportion of GWAS association signals demonstrate gene regulation effects [20], risk scores built on eQTL variants, known as transcriptomic risk scores (TRSs), are promising gene-based approaches that use gene expression information to identify trait-associated genes from GWAS. TRSs are significantly associated with a range of outcomes, including Amyotrophic Lateral Sclerosis [21], Alzheimer’s disease [22], and Crohn’s disease [23] based on observed gene expression data, as well as with ADHD symptoms [24], schizophrenia [25, 26], and major depressive disorder [24, 27] constructed with predicted gene expression. In addition, the combination of TRS with PRS improves risk prediction of several traits, including rheumatoid arthritis, height, body mass index or intelligence [24].

In the present study, we ran a multi-tissue TWAS on the latest GWAS-MA on ADHD performed so far [5], and for the first time used TWAS results to select a subset of signature genes per tissue and construct microarray-based TRSs in peripheral blood mononuclear cells (PBMCs), tested their association with ADHD and assessed whether the combination of PRS and TRS increases significantly the proportion of variance explained of ADHD over PRS alone, in subjects with ADHD and controls.

## Materials and methods

### Multi-tissue transcriptome-wide association study (TWAS)

TWAS was performed with S-PrediXcan (https://github.com/hakyimlab/MetaXcan) [28] using summary statistics from the largest GWAS-MA on ADHD to date in 38,691 individuals with ADHD and 186,843 controls [5], and SNP-weights of gene expression precomputed with the joint-tissue imputation (JTI) approach [29]. We used genetic variants with minor allele frequency (MAF) ≥ 0.01 and INFO score ≥ 0.80, and gene expression reference panels from GTEx v8 in 14 tissues, including whole blood, amygdala, anterior cingulate cortex, caudate basal ganglia, cerebellar hemisphere, cerebellum, cortex, frontal cortex, hippocampus, hypothalamus, nucleus accumbens basal ganglia, putamen basal ganglia, spinal cord cervical C1 and substantia nigra [30]. According to the GTEx webpage (https://gtexportal.org/home/samplingSitePage) both cortex and frontal cortex correspond to the same brain area, right cerebral frontal pole cortex, sampled and collected using different techniques. We considered default settings in S-Predixcan and linkage disequilibrium (LD) estimates from the European subset of the 1000 Genomes Phase 3 reference sample with the precalculated covariances. As TWAS results from different brain areas were highly correlated (r^2^ > 0.96 when considering genes nominally associated with ADHD), we applied Bonferroni correction considering the number of genes tested within each of the 14 expression reference panels separately to account for multiple testing.

Summary statistics from TWAS in DLPFC described in Demontis et al. 2022 were also used in the TRS analysis [5]. In brief, the reference panel was constructed using EpiXcan and expression data on DLPF of 924 samples with European ancestry from the PsychENCODE Consortium [31], and the S-PrediXcan method was used to integrate the ADHD GWAS meta-analysis summary statistics [5].

Enrichment analyses on gene-sets from the Molecular Signatures Database (MSigDB v6.2), including Gene ontology (GO), KEGG, Reactome, miRNA targets and GWAS Catalog, were performed on genes nominally associated with ADHD in each TWAS using a hypergeometric test with the GENE2FUNC module of FUMA and considering all genes from the TWAS as background [32]. Enrichment analyses results were corrected for multiple comparisons in each tissue considering each category separately using 5% False Discovery Rate (FDR).

#### Gene locus-level colocalization analysis

Gene locus-level colocalization probability (GLCP) for significant genes identified in TWAS was performed using fastENLOC and only genes with a GLCP ≥ 0.5 were considered further [33, 34]. First, we selected the genetic variants within 1 Mb upstream and 500 kb downstream from each of the 56 significant genes identified in TWAS with a *P* < 0.05 in the GWAS-MA of Demontis et al. [5]. These variants were fine-mapped to generate 95% credible sets, assuming one causal variant per locus, using the CAUSALdb pipeline (https://github.com/mulinlab/CAUSALdb-finemapping-pip#4; [35]) which includes three different fine-mapping tools, FINEMAP 1.3.1 [36], PAINTOR v3.0 [37] and CAVIARBF v.0.2.1 [38]. We used the recommended parameters of each tool and only variants selected by all three methods were considered. For these variants, Z-scores from the GWAS-MA on ADHD [5] were then converted to posterior inclusion probabilities using the *torus* software [39]. Finally, these data were colocalized with fastENLOC for the 14 GTEx v8 tissues included in the study [33]. Colocalization was performed using pre-computed GTEx multi-tissue annotations obtained from https://github.com/xqwen/fastenloc.

### Transcriptomic and polygenic risk scores

#### Participants and clinical assessment

TRSs and PRS were constructed in an in-house sample of 222 medication-naïve adult ADHD cases (59.45% male, mean age=34.03 years, s.d = 11.62) and 269 controls (57.25% male, mean age=36.6 years, s.d = 10.06). All subjects were from European ancestry, which was confirmed through principal component analysis (PCA) using genetic data. Clinical assessment was conducted by structured interviews and self-reported questionnaires as previously described [14], based in two steps: (i) assessment of ADHD diagnosis based on symptomatology using the Conner’s Adult ADHD Diagnostic Interview for DSM-IV (CAADID) and (ii) assessment of the severity of ADHD symptoms, the levels of impairment and the presence of comorbid disorders to increase the diagnostic accuracy with the Conners’ ADHD Rating Scale (CAARS), the ADHD Rating Scale (ADHD-RS), the Clinical Global Impression (CGI), the Wender Utah Rating Scale (WURS), the Sheehan Disability Inventory (SDS), and the Structured Clinical Interview for DSM-IV Axis I and II Disorders (SCID-I and SCID-II). Exclusion criteria were IQ < 70; a history or the current presence of a condition or illness, including neurologic, metabolic, cardiac, liver, kidney, or respiratory disease; a chronic medication of any kind; birth weight ≤1.5 kg; and other neurological or systemic disorders that might explain ADHD symptoms. All cases were evaluated and recruited prospectively from a restricted geographic area in a specialized out-patient program for adult ADHD at the Hospital Universitari Vall d’Hebron of Barcelona (Spain).

The control sample consisted of unrelated blood donors matched by sex with the clinical group. Individuals with ADHD symptomatology were excluded retrospectively from the control sample under the following criteria: (1) diagnosed with ADHD previously and (2) answering positively to the life-time presence of the following ADHD symptoms: (a) often has trouble in keeping attention on tasks, (b) usually loses things needed for tasks, (c) often fidgets with hands or feet or squirms in seat, and (d) often gets up from seat when remaining in seat is expected. The study was approved by the Clinical Research Ethics Committee (CREC) of Hospital Universitari Vall d’Hebron, methods were performed in accordance with the relevant guidelines and regulations and written informed consent was obtained from all subjects before inclusion in the study.

#### Transcriptomic risk scores

TRSs were constructed from transcriptomic profiles in PBMCs separated by a Ficoll density gradient method immediately after blood extraction. Total RNA was isolated using Qiazol Lysis reagent and the RNAeasy Midi Kit (QIAgen, Hilden, Germany). RNA integrity and concentration were assayed by 2100 Bioanalyzer (Agilent Technologies Inc., Santa Clara, CA, USA). RNA was retrotranscribed using the Ambion WT Expression Kit (Life Technologies, Carlsbad, CA, USA). The cDNA was subsequently fragmented, labelled, and hybridized with the GeneChip WT Terminal Labelling and Hybridization Kit (Affymetrix, Santa Clara, CA, USA). Samples were hybridized to the GeneChip Human Gene 1.1 ST 96-Array plate (Affymetrix), covering a total of 36,079 transcripts that correspond to 21,014 genes. The array processing and data generation were assessed using the Gene Titan Affymetrix microarray platform. Raw data were pre-processed as previously described [40]. In brief, data was processed with the Robust Multichip Analysis (RMA) algorithm from *OligoR* [41], sample outliers were removed using the *arrayQualityMetrics* [42] and transcript probes were filtered ending up with 19,004 probes corresponding to 18,055 unique genes. Microarray batch effects and non-biological experimental variation (RNA integrity number (RIN), age and gender) were adjusted for using the *empiricalBayesLM* algorithm included in WGCNA R package [43]. Raw data from this article is not publicly available because of limitations in ethical approvals and the summary data will be available upon request.

TRSs were calculated as the sum of the standardized expression of each gene weighted by its signed Z-score value from TWAS results on the different expression reference panels. TRSs per tissue were constructed by selecting genes under several TWAS *P*-value thresholds (Bonferroni, 0.001, 0.05, 0.1, 0.2, 0.3, 0.4, 0.5 and 1) and tested for association with ADHD using a logistic regression model in R, with sex, age, GWAS wave and the 10 first principal components based on GWAS data as covariates. For the best *P*-value threshold in each tissue, the empirical *P*-value was calculated by permuting the target phenotype 10,000 times and repeating the TRS analysis on each set of permuted phenotypes [44]. P*seudo*-R^2^ were calculated using the Lee’s formula [45] and considering an ADHD population prevalence of 5%. The effective number of independent tests was assessed with the Galwey method [46] considering Pearson correlation among TRSs from the best *P*-value threshold at each tissue, which resulted in 11 independent tissues out of 14. To account for multiple testing, we used the Sidák correction (*P*-value < 4.6e-03) for 11 independent tests. To discard an artificial inflation of the results due to the inclusion of different genes at the same genomic loci under the control of the same eQTL in the TRS construction, a sensitivity analysis was performed by calculating TRSs considering a single gene per locus: the one showing the lowest *P*-value in the TWAS at each genomic loci (defined by genes < 500 kb apart). Colocalization analyses were conducted using the same strategy described in the TWAS section, selecting genetic variants within a genomic window of 1 Mb upstream and 500 kb downstream from each of the genes in the best *P*-value threshold of TRSs associated with ADHD after multiple comparison corrections and sensitivity analyses.

#### Polygenic risk score

DNA samples were genotyped in two genotyping waves using Omni2.5 (*n* = 163) and Infinium™ Global Screening Array-24 v2.0 (*n* = 328) Illumina arrays. Polygenic scoring was conducted using the summary statistics from the largest GWAS-MA on ADHD in 38,691 individuals with ADHD and 186,843 controls [5], the PRS-CS software to generate posterior SNP effect sizes under continuous shrinkage (CS) priors to model LD between genetic variants (https://github.com/getian107/PRScs) [47]. The European subset of the 1000 Genomes Phase 3 reference was used to estimate LD and a global shrinkage parameter of phi = 1e-02 was considered. The PRS was generated using PLINK 1.09 software [48] and it was tested for association with ADHD using a logistic regression model, with sex, age, GWAS wave and the 10 first principal components based on GWAS data as covariates. The increment in *pseudo*-R2 was calculated using the Lee’s formula [45] and considering an ADHD population prevalence of 5%. Correlation between significant TRSs and PRS were calculated using the Pearson correlation coefficient. A likelihood ratio test with the *lmtest* R-package was used to compare the goodness of fit of the model that includes the PRS and covariates with the model that also includes the TRS.

## Results

### Transcriptome-wide association study

We performed a TWAS in ADHD using multiple brain tissues and whole blood expression reference panels and summary-level data from the largest GWAS-MA on ADHD so far in 38,691 cases and 186,843 controls [5, 30] (Supplementary Fig. S1). Overall, we tested 20,225 predicted genes across expression reference panels, ranging from 6213 to 11,473 depending on the tissue under study, representing at least 95% of the genes included in each expression reference panel (Supplementary Table S1). We identified a total of 4134 unique genes showing nominal association (*P* < 0.05) with ADHD in at least one tissue, including 2234 that were significant in more than one and 94 in all of them. These genes were enriched for genes previously associated with social interaction (e.g. regular attendance at a religious group, regular attendance at a gym or sports club or social communication problems), psychiatric disorders (e.g. autism spectrum disorder, schizophrenia or bipolar disorder) and body fat distribution, among others (Supplementary Table S2). Besides, analysis on miRNA target genes revealed significant enrichment of targets of miRNA-34b/c and miR-449 among genes differentially expressed in the cerebellum and of 14 mature miRNAs in cortex (Supplementary Table S3). No association with other categories from the MSigDB was found.

**Fig. 1:**
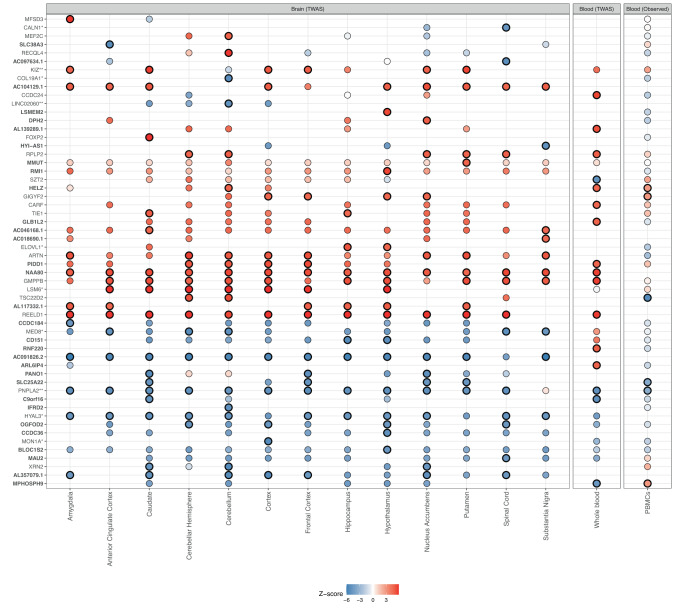
Observed and predicted (multi-tissue TWAS) differential expression for ADHD. Z-scores are plotted for the 56 genes significantly associated with ADHD in at least one of the studied tissues. Significantly associated genes are outlined in black (TWAS results with *P* < 8e-06, and observed expression in blood FDR < 0.05). The dots are color-coded based on the Z-scores of the genes, with white indicating a Z-score of 0, blue indicating a negative Z-score, and red indicating a positive Z-score. In the y axis novel genes that were not previously identified in the original ADHD GWAS or TWAS on the dorsolateral prefrontal cortex by Demontis et al., 2022 are highlighted in bold. * Genes previously reported in the GWAS and TWAS on ADHD by Demontis et al., 2022. ** Genes previously reported in the TWAS on ADHD by Demontis et al., 2022.

After Bonferroni correction, 56 unique genes in 28 independent loci (defined by genes > 500 kb apart) showed transcriptome-wide significance, of which 28 were significant in more than one tissue, all of them showing consistent direction of the effect (Fig. 1 and Supplementary Table S4). Of them, 8 genes were identified both in blood and at least one brain tissue, and 26 in at least two brain areas, being *NAA80* the only gene differentially expressed in all the studied tissues (Fig. 1 and Supplementary Table S4). From the genes identified in the TWAS, 31 were novel and 25 were previously associated with ADHD either by TWAS or GWAS in the study by Demontis et al. 2022 (Fig. 1, Supplementary Table S5 and Supplementary Fig. S2).

When comparing the predicted differential expression from TWAS with observed differential expression in PBMCs in our in-house sample, we found that 41 out of 56 genes identified in TWAS were available in our microarray analysis and from those, six were significantly differentially expressed. Out of the five genes differentially expressed in PBMCs and in at least one brain tissue, four showed consistent direction of effect (*HELZ, GIGYF2, SLC25A22* and *PNPLA2*), with *PNPLA2* and *HELZ* also differentially expressed in the whole blood TWAS and with consistent direction of effect (Fig. 1 and Supplementary Table S4). *TSC22D2* had discordant direction of effect between PBMCs and cerebellar hemisphere/cerebellum and *MPHOSPH9* between PBMCs and whole blood (Fig. 1 and Supplementary Table S4). Finally, colocalization analyses of the 56 genes identified in TWAS revealed 14 genes with a GLCP ≥ 0.5 in at least one of the studied tissues, four of them differentially expressed also in PBMCs with consistent direction of effect (*GIGYF2*, *HELZ*, *PNPLA2* and *SLC25A22*; Supplementary Table S6). *PNPLA2* was the most ubiquitous gene found colocalized in 9 tissues (GCLP range: 0.643– 0.896), followed by *REELD1* in 8 tissues (GCLP range: 0.615–0.818), and *LSM6* in 7 tissues (GCLP range: 0.724–0.844; Supplementary Table S6).

### Transcriptomic risk scores

TRSs based on multi-tissue TWAS results were constructed at different significance thresholds using expression data from PBMCs in an in-house sample of 222 subjects with ADHD and 269 controls (Supplementary Fig. S1). We found strong evidence of association in brain, with TRSs based on TWAS from 11 out of 13 brain tissues significantly associated with ADHD status after computing the empirical *P*-values (free from inflation due to overfitting) being cortex the most significant one (*P*_empirical_ = 1e-04; Table 1 and Supplementary Fig. S3).

**Table 1:** Models considering Transcriptomic Risk Scores (TRSs) or TRSs in combination with Polygenic Risk Scores (PRS).

	N*	TRS					PRS+TRS					
							PRS**		TRS			Likelihood ratio test *P*-value
		Best *P*-value threshold^a^	Estimate	*P*-value	Empirical *P*-value	*Pseudo*-R^2^	Estimate	*P*-value	Estimate	*P*-value	*Pseudo*-R^2^	
Amygdala^b^	68	0.001	0.28	4.3E-03	3.5E-03	0.013	0.34	6.9E-04	0.29	3.2E-03	0.034	2.8E-03
Anterior Cingulate Cortex	71	0.001	0.25	8.9E-03	9.7E-03	0.011	0.36	3.7E-04	0.28	3.4E-03	0.033	3.0E-03
Caudate^b^	12	BF	0.30	1.9E-03	1.5E-03	0.016	0.34	7.0E-04	0.31	1.5E-03	0.036	1.2E-03
Cerebellum	19	BF	0.23	0.019	0.017	0.009	0.33	8.0E-04	0.23	0.016	0.029	0.015
Cortex^b^	10	BF	0.44	1.5E-05	1.0E-04	0.032	0.34	8.1E-04	0.44	1.3E-05	0.052	7.1E-06
Dorsolateral Prefrontal Cortex^bc^	21	BF	0.40	6.6E-05	9.9E-05	0.028	0.32	1.5E-03	0.39	1.1E-04	0.046	6.9E-05
Frontal Cortex^b^	87	0.001	0.36	2.4E-04	4.0E-04	0.023	0.33	9.1E-04	0.37	2.3E-04	0.042	1.7E-04
Hippocampus	6	BF	0.22	0.020	0.021	0.009	0.34	6.4E-04	0.24	0.013	0.029	0.012
Nucleus accumbens	14	BF	0.27	6.5E-03	6.9E-03	0.012	0.33	1.1E-03	0.26	7.9E-03	0.031	7.2E-03
Putamen^bd^	10	BF	0.36	3.2E-04	5.0E-04	0.022	0.30	2.7E-03	0.34	9.1E-04	0.038	7.1E-04
Spinal cord	7	0.001	0.26	7.9E-03	8.4E-03	0.012	0.34	6.1E-04	0.27	5.2E-03	0.032	4.7E-03
Substantia nigra	1968	0.4	-0.21	0.028	0.033	0.008	0.33	1.0E-03	-0.21	0.031	0.027	0.030

^**^PRS-only model results: Estimate= 0.3295; *p*-value = 9.41E-04, Pseudo-R^2^ = 0.0189. ^*^Number of genes included in the TRS.

^a^BF: Bonferroni significance threshold.

^b^TRSs significantly associated with ADHD after the multiple testing correction of Sidák.

^c^TWAS from Demontis et al. 2022.

^d^Not significant after sensitivity analysis.

**Fig. 2:**
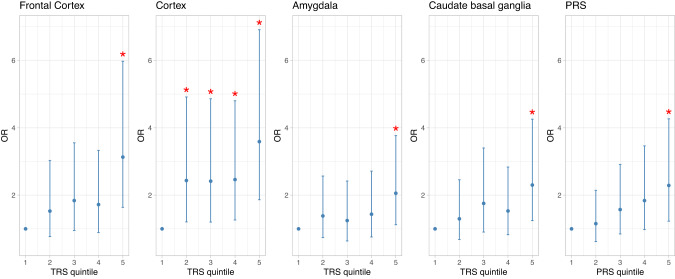
Quintile plot of odds ratios for PRS and TRS. Odds ratios (OR) with 95% confidence intervals are shown for PRS and TRSs that overcome multiple comparison corrections and sensitivity analysis using the first quintile as baseline.

**Fig. 3:**
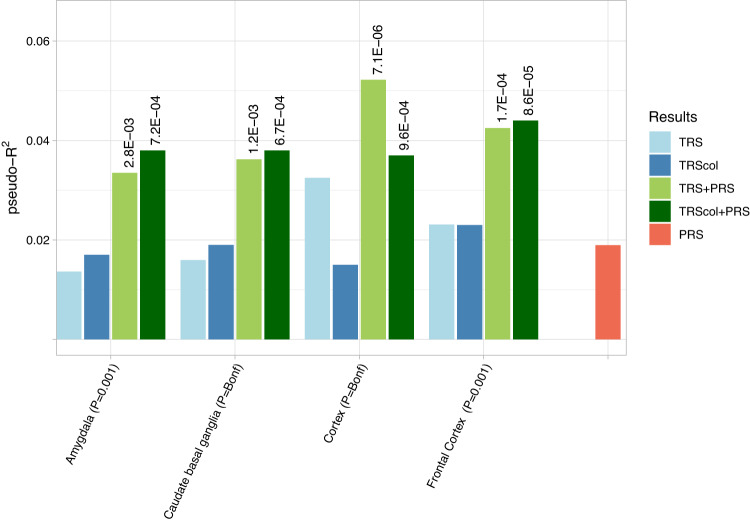
Proportion of variance explained by TRSs significantly associated with ADHD and by PRS. Pseudo*-*R^2^ (in the y-axis) is presented for each TRS, TRS restricted to colocalized genes (TRS_col_), the PRS and the model that combines both scores (TRS + PRS or TRS_col_ + PRS). Values in brackets indicate the best *P*-value threshold for a given tissue (P). Likelihood ratio test *P*-values for TRS and PRS vs. PRS-only model comparisons are given above the bars. Further statistical details can be found Supplementary Table S9.

Although significant associations with ADHD were observed across the different TWAS *P*-value thresholds in most of the brain areas, there was clear evidence of increased proportion of variance explained by TRSs as lower *P*-value thresholds were used (Supplementary Fig. S3). After correction for multiple comparisons, TRSs remained significantly associated with ADHD when constructed on TWAS from five brain tissues, including cortex (*P*_empirical _= 1.0e-04, *pseudo-*R^2^ = 0.032), frontal cortex (*P*_empirical _= 4.0e-04, *pseudo-*R^2^ = 0.023), putamen (P_empirical _= 5.0e-04, *pseudo-*R^2^ = 0.023), caudate basal ganglia (*P*_empirical _= 1.5e-03, *pseudo-*R^2^ = 0.016) and amygdala (*P*_empirical _= 3.5e-03, *pseudo-*R^2^ = 0.014), with subjects with ADHD having a significantly higher ADHD-TRS than controls in all of them (Table 1 and Supplementary Fig. S4). Associations remained significant in the sensitivity analyses considering only the most significant gene per locus in the TRS construction, with the exception of the TRS based on TWAS results in putamen (Supplementary Table S7 and Fig. S5). The quintiles of the remaining TRSs showed the expected trend of higher ADHD odds for individuals in higher quintiles (Fig. 2) and positive correlations were found between the four TRSs (corrected *P* < 7.1e-04 and 0.22≤ r  ≤ 0.62) (Supplementary Fig. S6). Out of the 112 genes included in at least one of these TRSs, three were used in all four: *GMPPB, PLK1S1* and *PNPLA2* (Supplementary Table S8). Despite the proportion of variance explained for the TRSs being in line with that of PRS (Estimate = 0.3295, *P* = 9.4e-04, *pseudo-*R^2^ = 0.019, Fig. 3), both scores were not correlated in any of the tissues with significant results after the sensitivity analyses (*r* ≤ -0.02; Supplementary Fig. S6) and combining TRSs and PRS improved the fit of the model over PRS alone (*P* < 0.03), with TRSs from cortex showing the best results and reaching a *pseudo-*R^2^ of 0.052 in the combined model (*P* = 7.1e-06, Table 1 and Fig. 3).

We also constructed TRSs restricted to colocalized genes (TRS_col_) for the TRSs significantly associated with ADHD after multiple comparison corrections and sensitivity analyses. We found that, despite reducing the number of genes included, the association signal remained in all four tissues and that the predictive performance improved for TRS_col_ in three of them, amygdala, caudate basal ganglia and frontal cortex (Supplementary Table S9). Interestingly, out of the 24 genes included in at least one of these TRS_col_, three genes were included in three out of the four analyses: *LSM6*, *PIDD1* and *PNPLA2*, with consistent direction effects across tissues (Supplementary Table S8). In line with the results from TRSs calculated with all genes, the combination of TRS_col_ with PRS improved the fit of the model over PRS alone for all four tissues (*P* < 9.59e-04; Fig. 3 and Supplementary Table S9).

Finally, to assess the robustness of our results further, we used TWAS results from DLPFC [5] on a larger reference panel from the PsychENCODE Consortium [31]. TRS from DLPFC was also significantly associated with ADHD (*P*_empirical_=9.9e-05, *pseudo-*R^2^ = 0.028), remained significant in the sensitivity analysis considering only the most significant gene per locus (Supplementary Table S7), and combined with PRS improved the fit of the model over PRS alone (*P* = 6.9e-05), reaching a *pseudo-*R^2^ of 0.046 in the combined model (Table 1).

## Discussion

To our knowledge, this is the first study to construct TRSs for ADHD based on observed expression data. We undertook TWAS on ADHD using the latest ADHD GWAS-MA summary statistics and 14 expression reference panels across a range of brain tissues and whole blood to prioritize genes and construct transcriptome-based risk scores for the disorder [5, 30]. Given that a substantial proportion of GWAS hits demonstrate gene regulation effects [20], risk scores based on eQTL variants integrate biological information for disease prediction, link genetic associations to biological disease mechanisms and provide an additional layer of biological interpretability.

We found 56 genes showing transcriptome-wide significant association with ADHD, of which 31 did not overlap with previously described GWAS loci or TWAS results by Demontis et al. [5]. The variability observed between studies could be mainly due to differences in the tissues and methods used to construct the expression reference panel, as Demontis et al. used a different eQTL reference panel in DLPFC from the PsychENCODE Consortium [31], and we used GTEx v8 data on 14 tissues based on JTI methodology, to exploit the power of multi-tissue transcriptomes to improve prediction accuracy. Among the new genes identified, *NAA80*, associated with ADHD in all expression reference panels, encodes an actin-specific N-acetyltransferase that may play a role in excitatory synapses, which is consistent with alterations in the reorganization of synaptic actin described in neurodevelopmental disorders [49]. *PNPLA2* was transcriptome-wide significant in all the expression reference panels but substantia nigra and differentially expressed in the PBMCs with consistent direction of effect. It encodes a lipase related with obesity, highly comorbid with ADHD [50], and was recently pointed as one of the most high-confidence causal genes for ADHD [17]. Other interesting transcriptome-wide significant signals included several long non-coding RNA, a group of regulatory RNA involved in neural differentiation and synaptic plasticity that have been related with psychiatric disorders [51, 52], or target genes for miRNA-34, previously associated with ADHD [53]. This miRNA family participates in neuronal differentiation and synaptogenesis [54] and is among the most upregulated miRNAs during dopaminergic differentiation [55].

We selected a subset of relevant genes from TWAS results and constructed TRSs using microarray expression data in PBMCs from 222 individuals with ADHD and 269 controls. TRSs based on TWAS results from most of the brain tissues were associated with ADHD, with individuals with ADHD carrying a higher burden of TRS than controls. In contrast, no association was found when the TRS was constructed based on TWAS results in whole blood, which suggests that the performance of the TRS is optimized when selecting genes from expression reference panels in relevant tissues for the disorder. This is likely due to the eQTL tissue specificity previously described [56] and is in line with our findings where the TRSs that surpassed multiple comparison corrections and sensitivity analyses were constructed from expression reference panels in four brain areas associated with ADHD, namely cortex, frontal cortex, caudate basal ganglia and amygdala [57–59].

Genes included in the best-performing TRSs provide additional information to prioritize candidates for further investigation of biological mechanisms underlying ADHD. For example, all TRSs associated with ADHD include three genes, *PNPLA2, PLK1S1* and *GMPPB*, previously associated with ADHD and/or other neurodevelopmental disorders [17, 60–62]. Of them, *PNPLA2*, already discussed as one of the top hits in the multiple-tissue TWAS, is the only gene with a high colocalization score in three out of the four tissues studied, and seems to play an important role in the TRS_col_ of amygdala, caudate basal ganglia and frontal cortex, which points it as one of the most promising candidate genes. Besides, we also highlight other genes with high colocalization scores in different tissues: the *GIGYF2* gene, significantly associated with ADHD across the lifespan [63], which contributes to the TRS_col_ from both cortex and frontal cortex, the *SLC25A22* gene, which encodes a glutamate transporter with strong expression in the developing brain, that adds important weight to the TRS_col_ from caudate basal ganglia and frontal cortex, and *CKS2*, a cyclin-dependent kinase involved in the control neuronal differentiation [64], which contributes to the TRS_col_ from the amygdala. Interestingly, according to the GWAS catalog genetic variants in these genes and others included in TRS_col_ (i.e. *CTNNB1, COPA, CCDC71* and *BLOC1S2*) have been associated with psychiatric disorders (e.g. schizophrenia, externalizing behavior, smoking initiation, autism spectrum disorder, anorexia nervosa, depression and anxiety disorder), cognitive function (e.g. intelligence, educational attainment and mathematical ability) or ADHD comorbid somatic traits like obesity or extreme body mass index, suggesting a potential importance of these genes in the context of ADHD and its comorbid conditions.

For most of the brain tissues, the TRSs constructed under stricter TWAS *P*-value thresholds showed clear evidence of better performance and stronger associations with ADHD, a pattern similar to the one observed for TRS in amyotrophic lateral sclerosis based on observed expression data [21]. This contrasts with the pattern of association found for PRSs or imputed gene expression-based risk scores, where the variance explained tends to increase as more relaxed *P*-value thresholds are used [24, 26]. These different patterns could result from methodological limitations in TWAS that hamper the statistical power of TRSs from observed gene expression, especially when more genes with weaker association signals are included in the analysis. These could include noisy beta estimates in TWAS due to the limited sample size of both GWAS-MA on ADHD and GTEx v8 reference panels [5, 30] , or false positive associations in the TWAS due to pleiotropy or linkage disequilibrium. Also, TRSs-based on observed expression data may reflect a dynamic layer of biological regulation that could explain the difference found. While using predicted expression data provides an accurate estimate of the genetic risk conferred via cis-regulated gene expression, TRSs constructed on observed expression datasets may be also attributable to other influences including trans-acting genetic effects or environmental effects and may provide a closer connection to the disorder than standard PRSs or TRSs calculated on imputed gene expression levels. This is consistent with findings showing that a substantial proportion of gene expression heritability may not result from common cis-eQTL SNPs, but rather stem from trans-variants which may act predominantly in a tissue-specific manner, and points to the need for further studies on the trans-regulatory landscape [65].

In agreement with a previous study in depression [66], TRSs were uncorrelated with genome-wide PRS. This lack of correlation may highlight that TRSs based on observed gene expression data capture more information than cis-eQTL genetic risk variants, such as trans-eQTL, environment factors or epigenetics, as well as interaction effects between genes and environment, among others. In addition, compared with PRS-only models, models combining PRS and TRSs provided substantial improvement in model fit for ADHD, which supports that gene expression explains additional phenotypic variance for the disorder than PRSs and is consistent with the complementary predictive potential of genetic and transcriptomic signatures [24].

Apart from TWAS, other methods have been designed to prioritize likely causal genes by combining genomic, transcriptomic, and other regulatory and functional information including colocalization methods, that use a Bayesian framework to infer whether a regulatory SNP is also responsible for the association with a trait of interest, or summary-based Mendelian randomization (SMR), that combines GWAS and eQTL data to prioritize target genes with evidence for causal or pleiotropic effects. In order to narrow down the number of genes identified by TWAS and included in the TRS analyses, we assessed colocalization and found that the signal for 14 out of the 56 genes identified in the TWAS was supported by the colocalization analyses. This low convergence between TWAS and colocalization signals is consistent with other studies [34] and may result from several factors including failure to identify either the phenotype-SNP association or the expression-SNP association, given the relatively limited sample size of both GWAS-MA on ADHD and GTEx v8 reference panels [5, 30], especially for brain areas. Also, colocalization signals may arise from direct genetic effects, while TWAS signals may result from complex interactions between multiple genes and genetic variants [33]. When restricting best-performing TRSs to the colocalized genes, despite a reduction of at least the 70% in the number of genes included, the association signal remained and even became stronger for amygdala, caudate basal ganglia and frontal cortex. These results are in line with previous studies [21, 23] and point to the high specificity of the colocalization approach [33].

The results of the present study, however, should be interpreted in the context of several strengths and limitations: (i) Due to linkage disequilibrium, a single genetic variant might point to several TWAS associations in the same locus. For that reason, sensitivity analysis using only the most significant gene in each locus was performed to discard artefactual inflation in the TRS analysis. However, considering that genes located in the same region are not necessarily involved in the same biological processes and given the difficulty to determine which ones really contribute to the phenotype, enrichment analysis were performed including all significant genes from TWAS, which could have potentially biased these results. (ii) In this study, the PRS failed to approach the performance of the two best-performing TRSs (from cortex and frontal cortex), which suggests that TRSs may potentially outperform PRSs and provide a closer physiological picture of the disorder; (iii) While TRSs differences may reflect distinct molecular pathways captured by each of the tissues considered, the variability in sample size between the expression reference panels may limit our ability to compare TRSs results across tissues. Besides, in the present study we used multiple-tissue TWAS. Although this method shows improved prediction over single tissue approaches and it underscores specific genes overlapping between tissues [29], additional approaches are required to identify tissue-specific expression profiles; (iv) We found significant correlation between the TRSs associated with ADHD, probably, in part, because the different brain areas from which they were constructed are both functionally and structurally connected. However, selecting genes for the construction of TRSs based on multiple-tissue TWAS results, where information is borrowed across transcriptomes of different tissues, may also contributed to artificially inflate these correlations; (v) The positive results obtained for TRSs capturing expression in brain areas implicated in ADHD but not in whole blood suggests that the relevance of the tissue to the outcome may also influence the predictive performance of the TRS; (vi) Although TRS constructed on real expression datasets may provide a closer connection to the disorder and may capture gene expression within a range of contexts, they may be influenced by confounding factors such as gender, age, comorbid disorders or medication. We frequency sex-matched ADHD cases and controls and restricted the clinical sample to ADHD medication-naïve adult subjects, which is a major strength of our study design that may allow us to identify transcriptomic signatures that might be neglected by broader study designs. We cannot discard residual confounding by other factors not available. In the same line, observed differential expression associated with ADHD may reflect both a gene’s causal role in the disorder or be consequence of the disorder itself. However, given that genetically-inferred differential expression from TWAS may not be susceptible to reverse causation, we think that most genes included in our TRSs are more prone to it because of the disorder rather than consequence; (vii) Further studies considering low frequency and rare variants and using more unbiased profiling methods, such as RNA sequencing techniques, may allow the inclusion of novel and low abundance transcripts and relevant genes to improve the predictive power of TRS approaches. In addition, as resources used for eQTL mapping expand in sample size and integrate additional regulatory and epigenetic data, we expect TRS performance to improve. (viii) Finally, longitudinal studies will be required to disentangle the performance of TRSs across the lifespan and their role on the remittent and/or persistent form of the disorder.

In conclusion, we found association between ADHD and TRSs in PBMCs constructed using TWAS results from multiple brain areas implicated in the disorder, showing that individuals with ADHD carry a higher burden of TRSs than controls. TRSs combined with PRS increased significantly the proportion of variance explained of ADHD over genome-wide PRS alone, which points to the complementary predictive potential of genetic and transcriptomic signatures and support that integrating biological information may benefit standard PRS prediction approaches. Through this approach that leverages GWAS summary statistics, multi-tissue cis-eQTL reference panels and target sample gene expression data we underscore the potential of utilizing transcriptomic information to improve risk prediction and provide deeper insight into the molecular mechanisms underlying ADHD.

### Supplementary information

Supplementary Figures

Supplementary Tables
