# Supplementary material for: Transcriptomic risk scores for attention deficit/hyperactivity disorder
Source: Mol Psychiatry. 2023 Aug 3;28(8):3493–502. doi: 10.1038/s41380-023-02200-1 (PMC10618083; doi:10.1038/s41380-023-02200-1)
Supplement: Supplementary file 1 — Supplementary Figures [file 41380_2023_2200_MOESM1_ESM.docx]

**Supplementary information**

**Transcriptomic risk scores for attention deficit/hyperactivity disorder**

**Table of content**

**Supplementary Figure S1.** Study design **………….…………………………………………… 1**

**Supplementary Figure S2.** Miami plots representing the TWAS results in multiple brain tissues and whole blood **.……………………………………………………………………………....... 2**

**Supplementary Figure S3.** Barplots showing the proportion of variance (pseudo-R2) of ADHD explained by the TRSs constructed at different TWAS P-value thresholds in multiple tissues **… 9**

**Supplementary Figure S4.** Density plots from TRSs associated with ADHD that overcome multiple comparison corrections **………………………………………………………...…….. 10**

**Supplementary Figure S5.** Comparison of TRSs effect sizes before and after sensitivity analysis.

**……………………………………………………………………..…..………………….…... 11**

**Supplementary Figure S6.** Heatmap representing correlation between TRSs and PRS **……... 12**


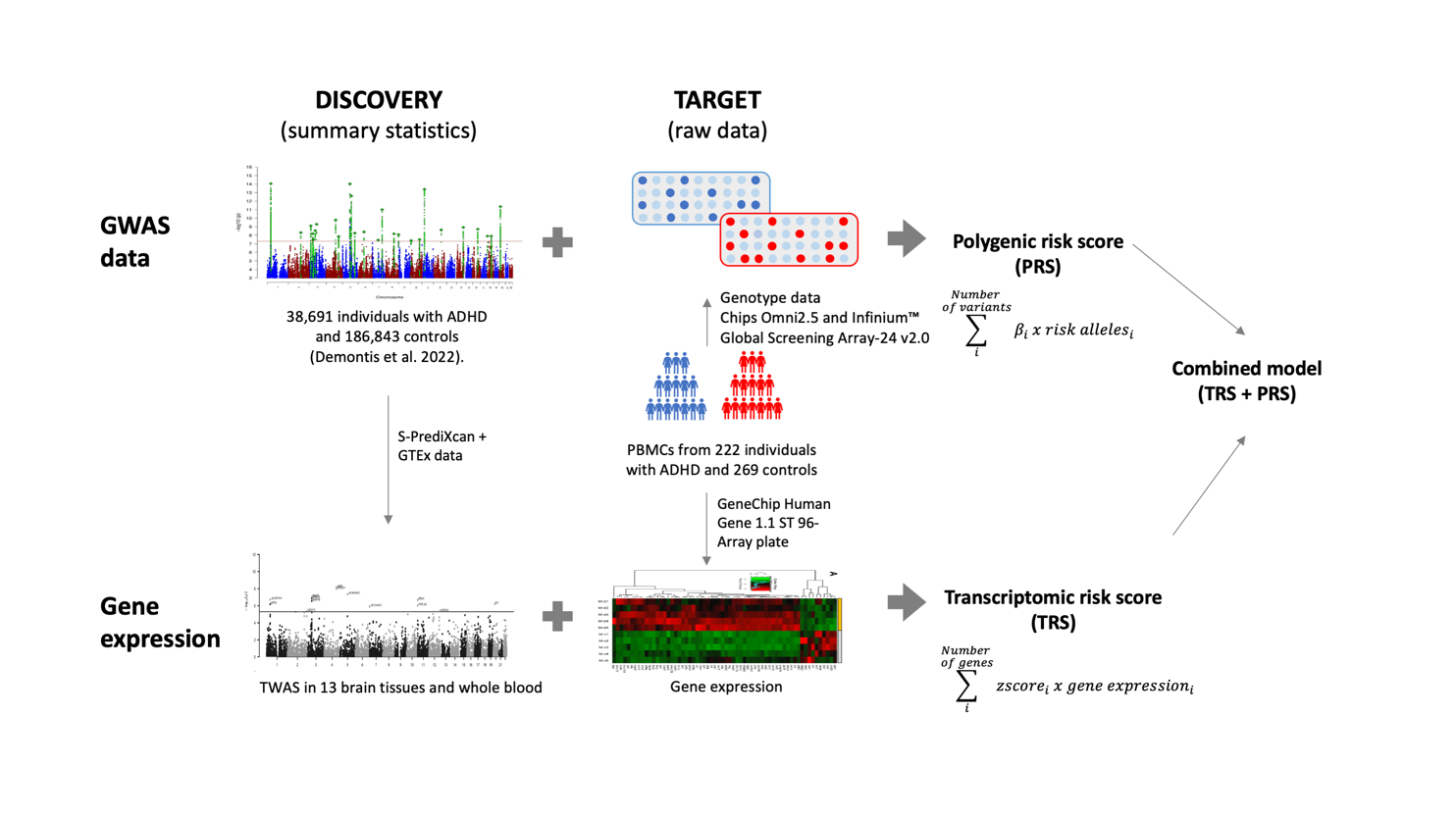


**Supplementary Figure S1**. **Study design.** Description of the discovery and target samples used and the steps followed in the construction of the polygenic and transcriptomic risk scores. ß: GWAS effect size; z-score: TWAS z-score.

Amygdala


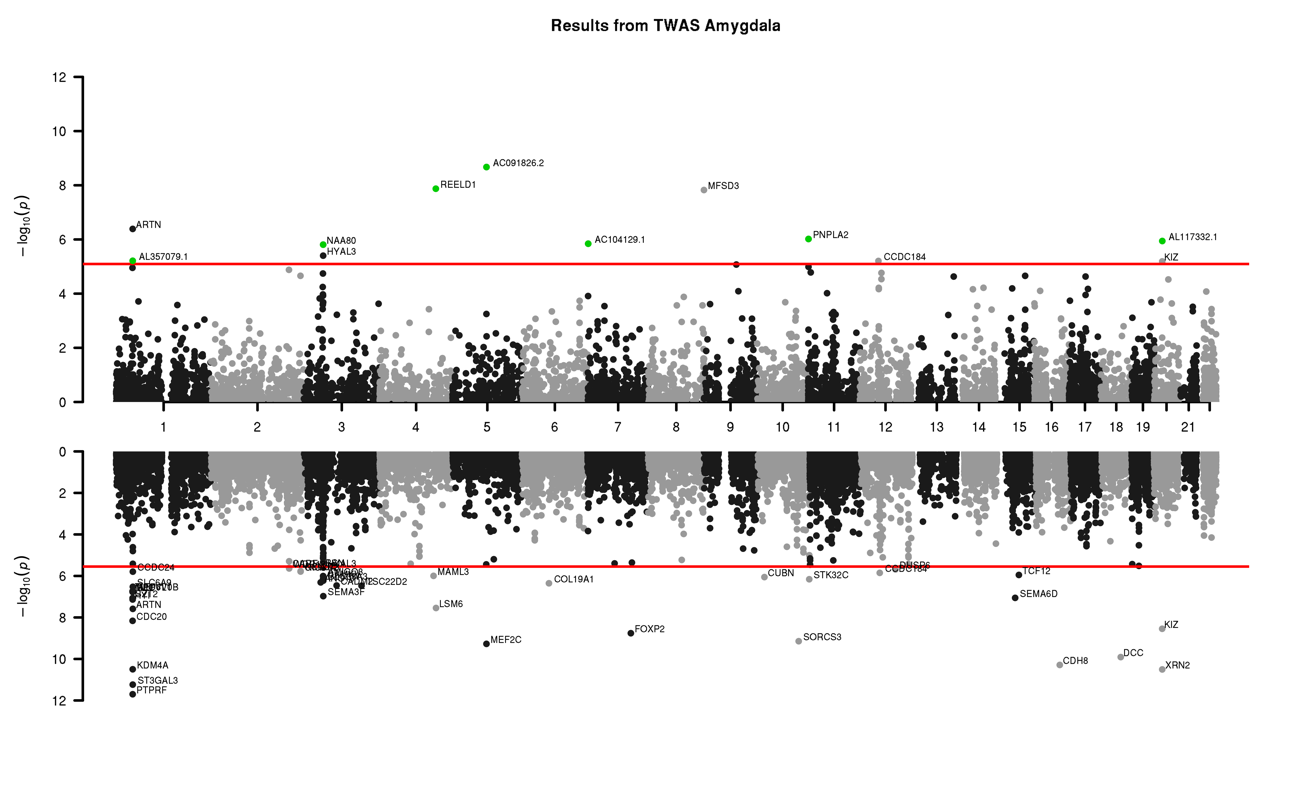


Anterior Cingulate Cortex


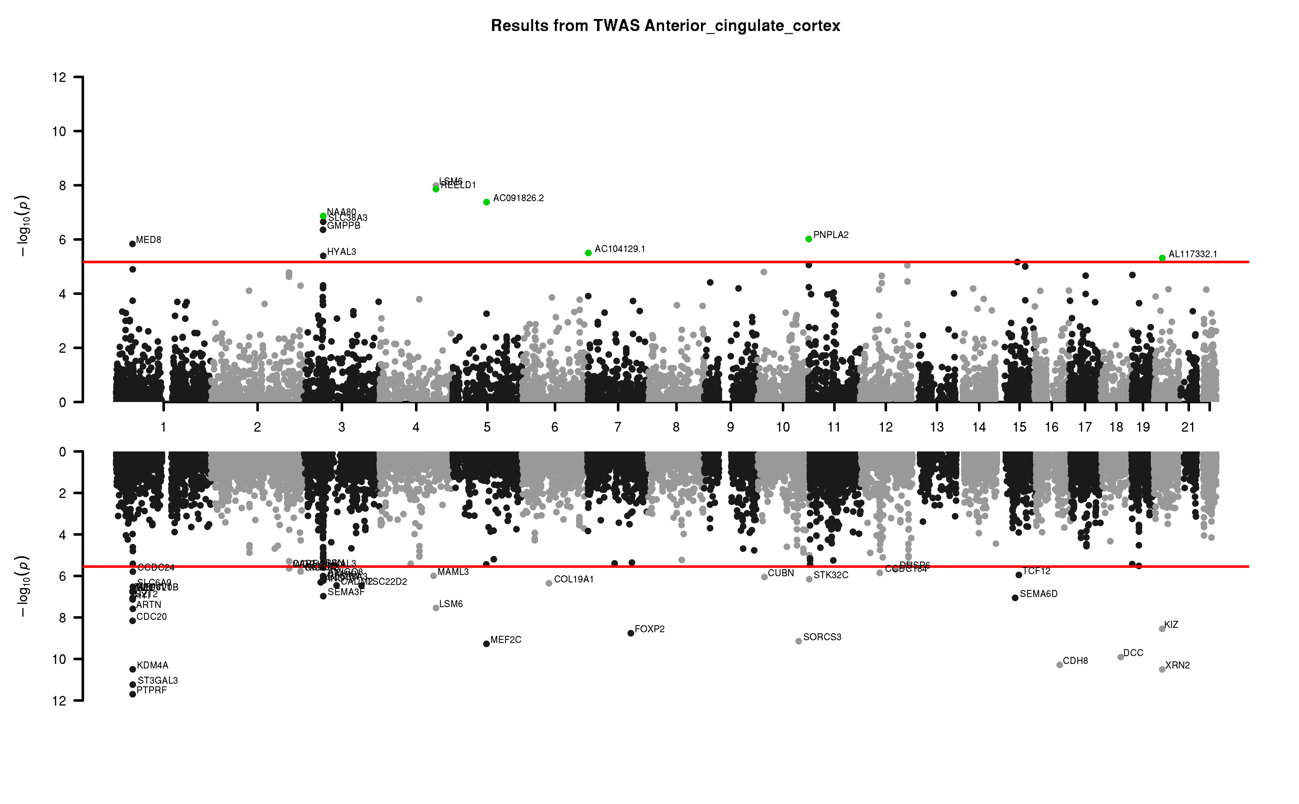


Caudate Basal Ganglia


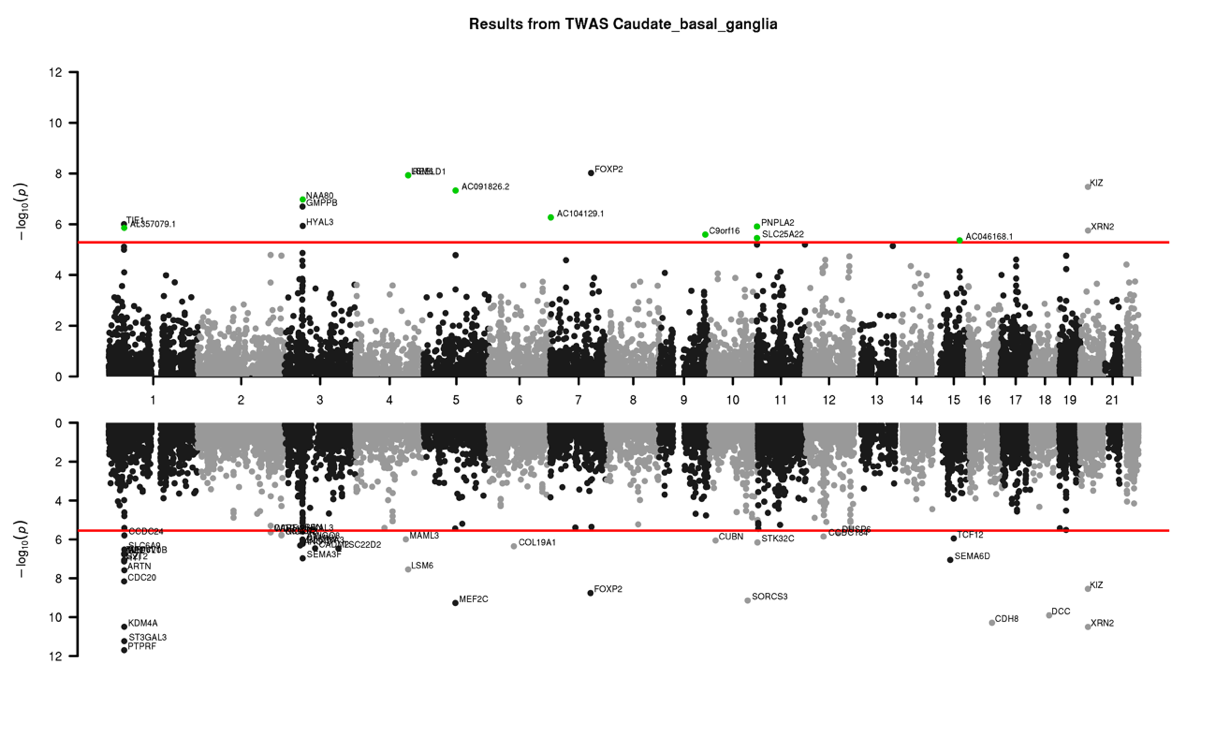


Cerebellar Hemisphere


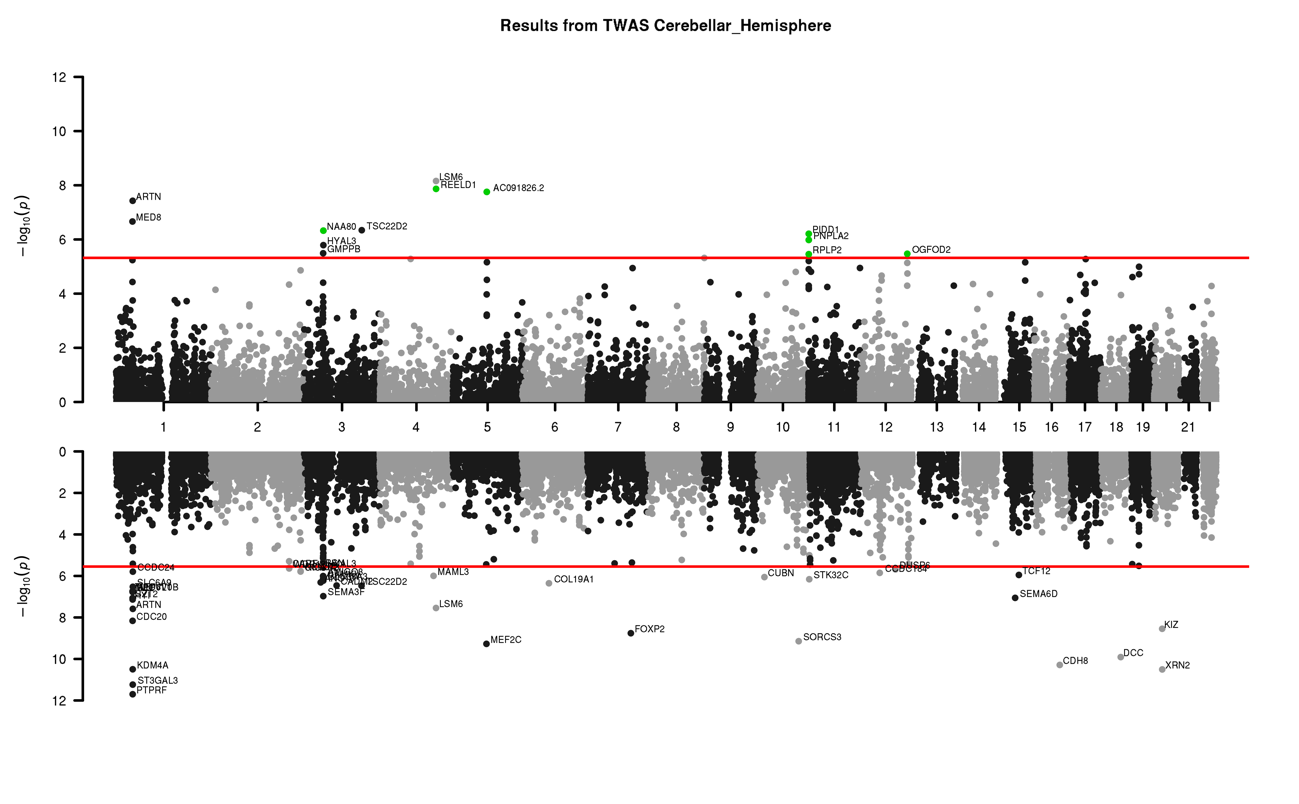


Cerebellum


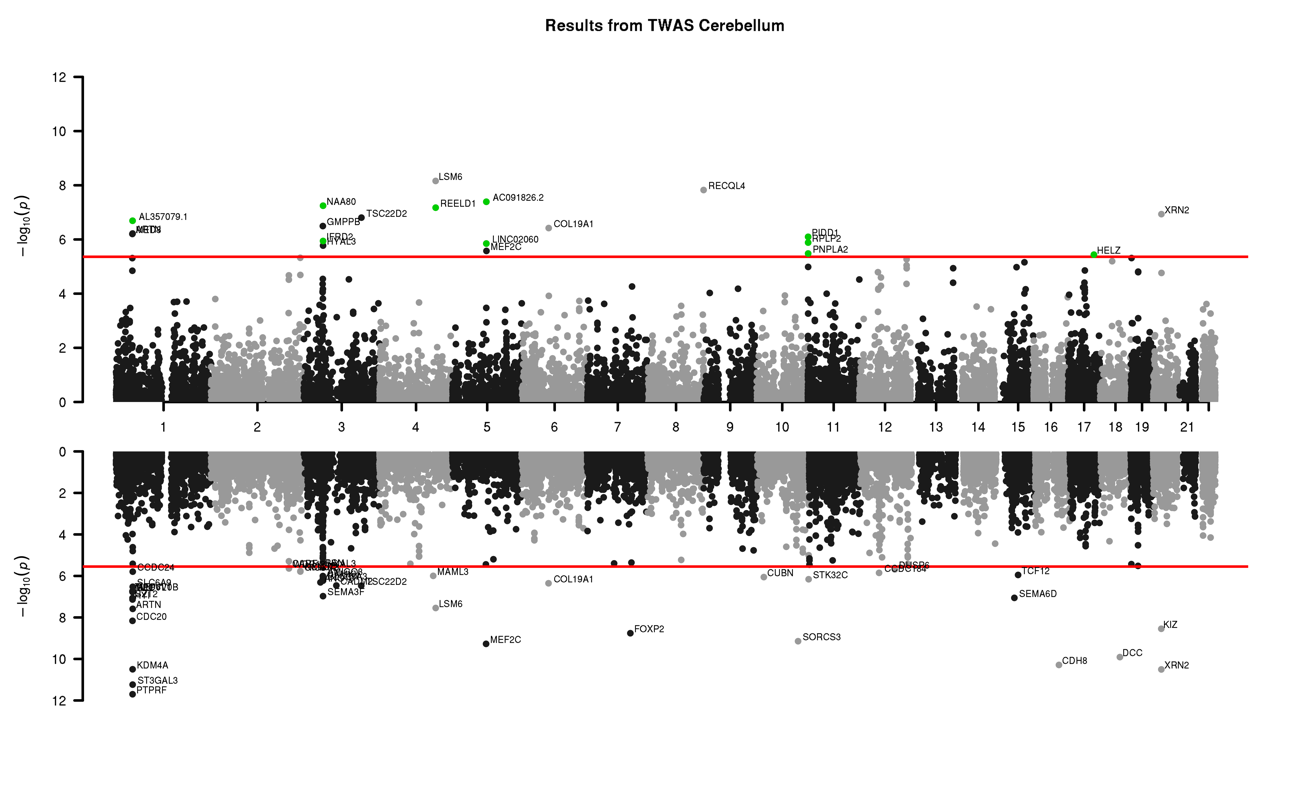


Cortex


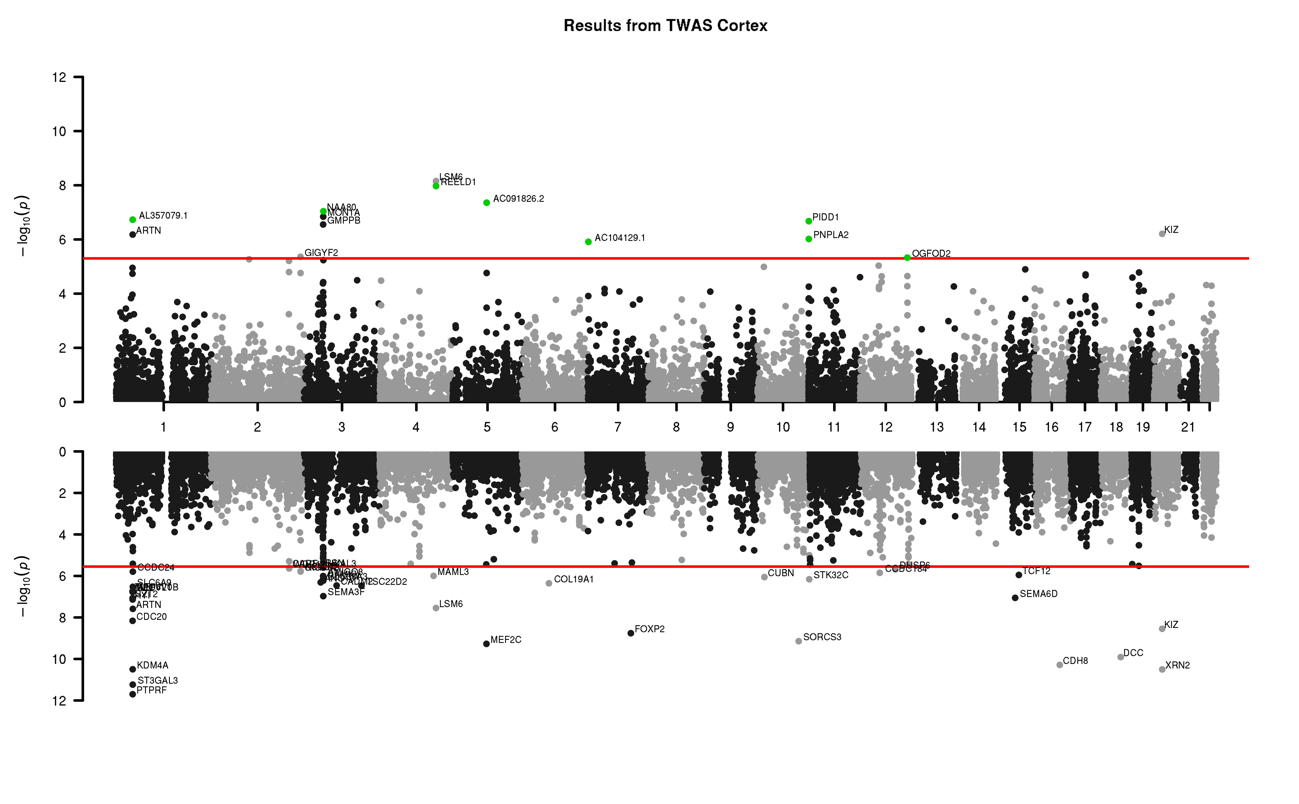


Frontal Cortex


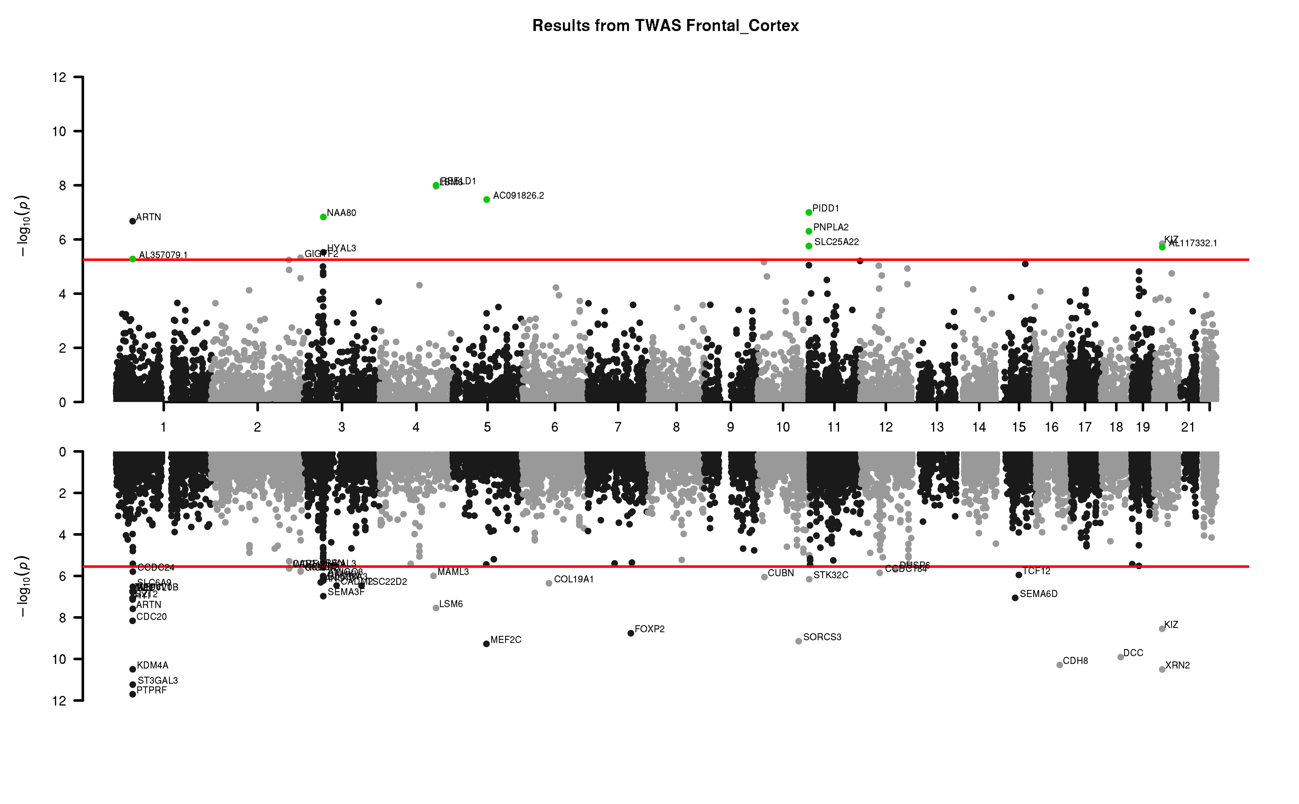


Hippocampus


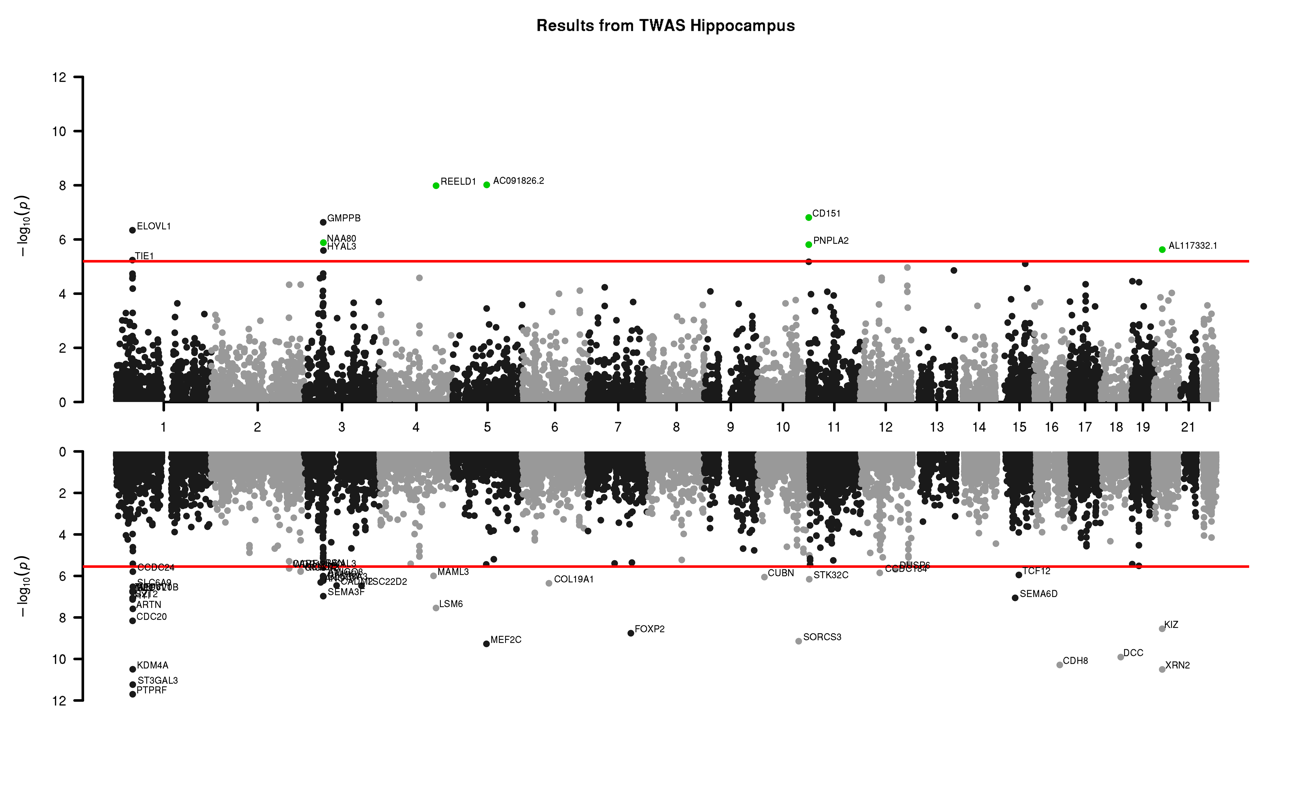


Hypothalamus


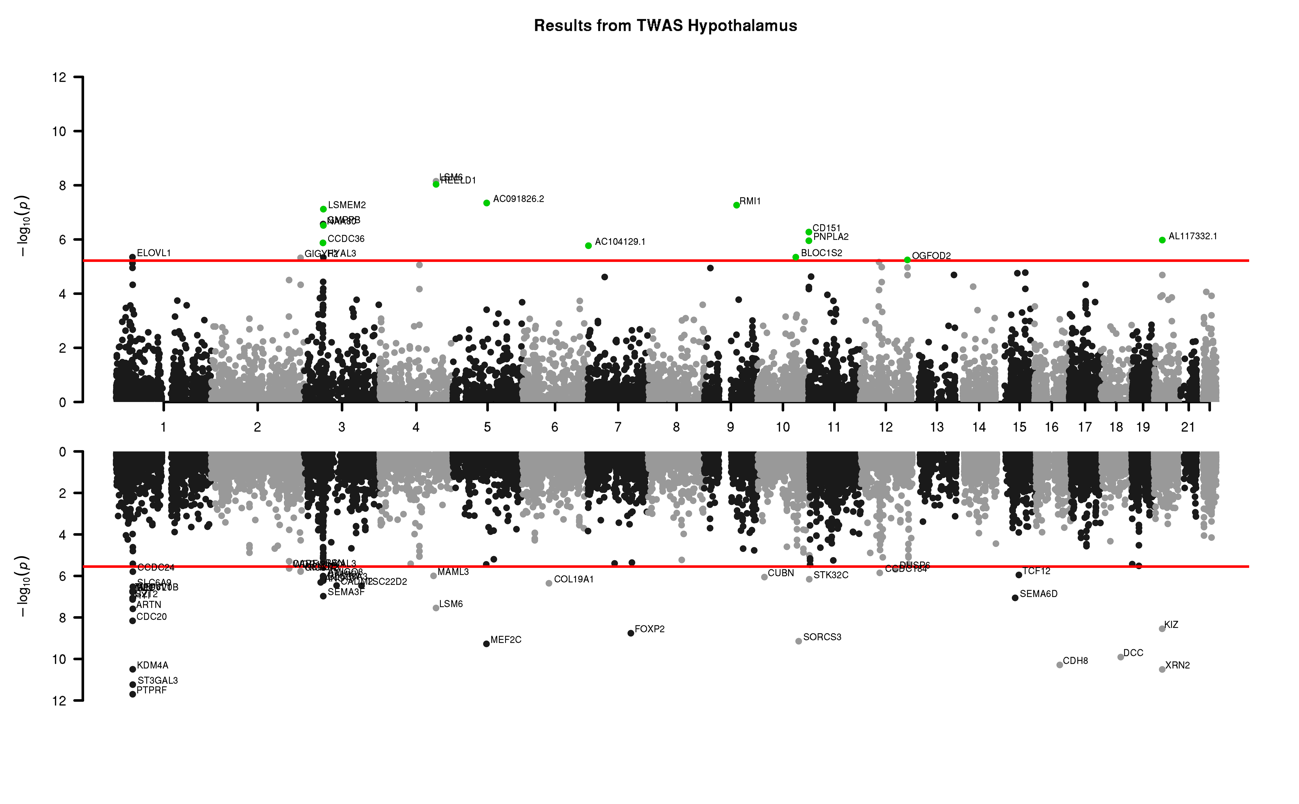


Nucleus Accumbens


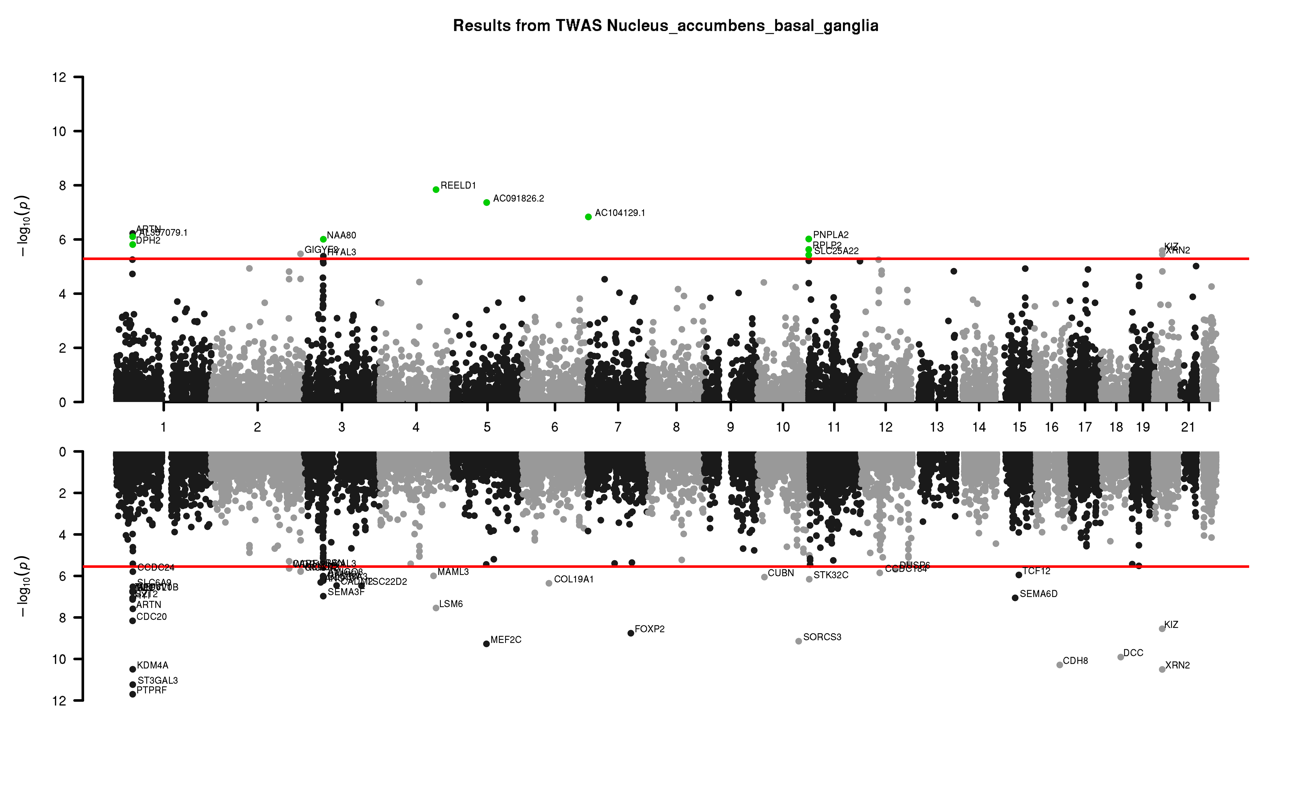


Putamen


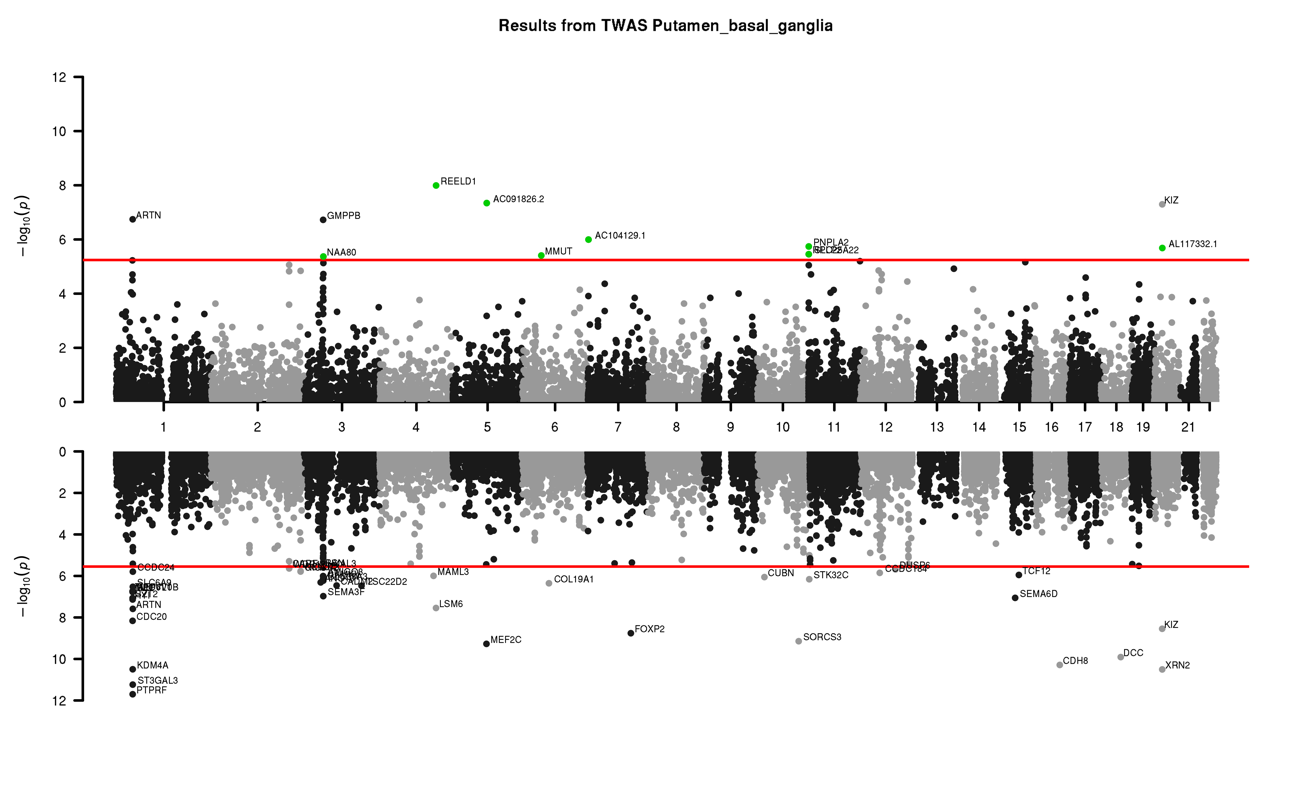


Spinal cord


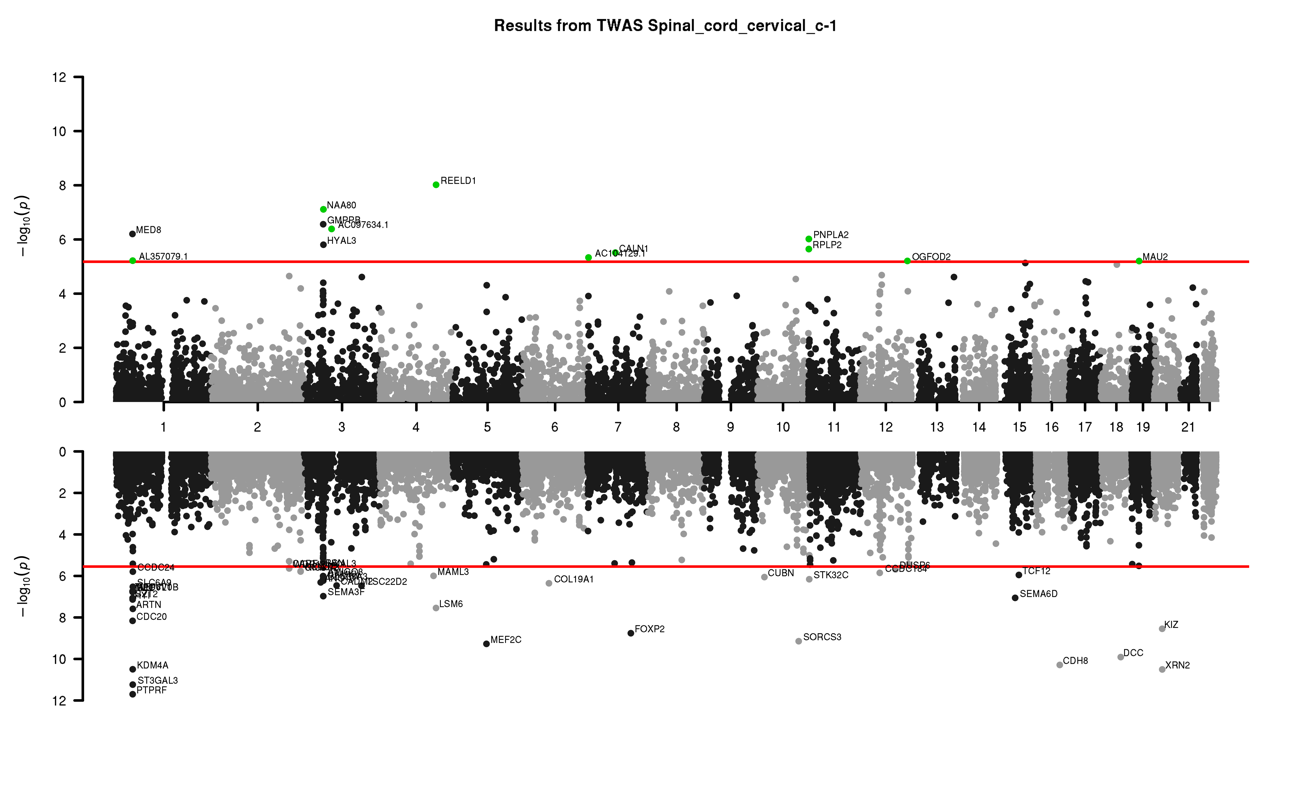


Substantia Nigra


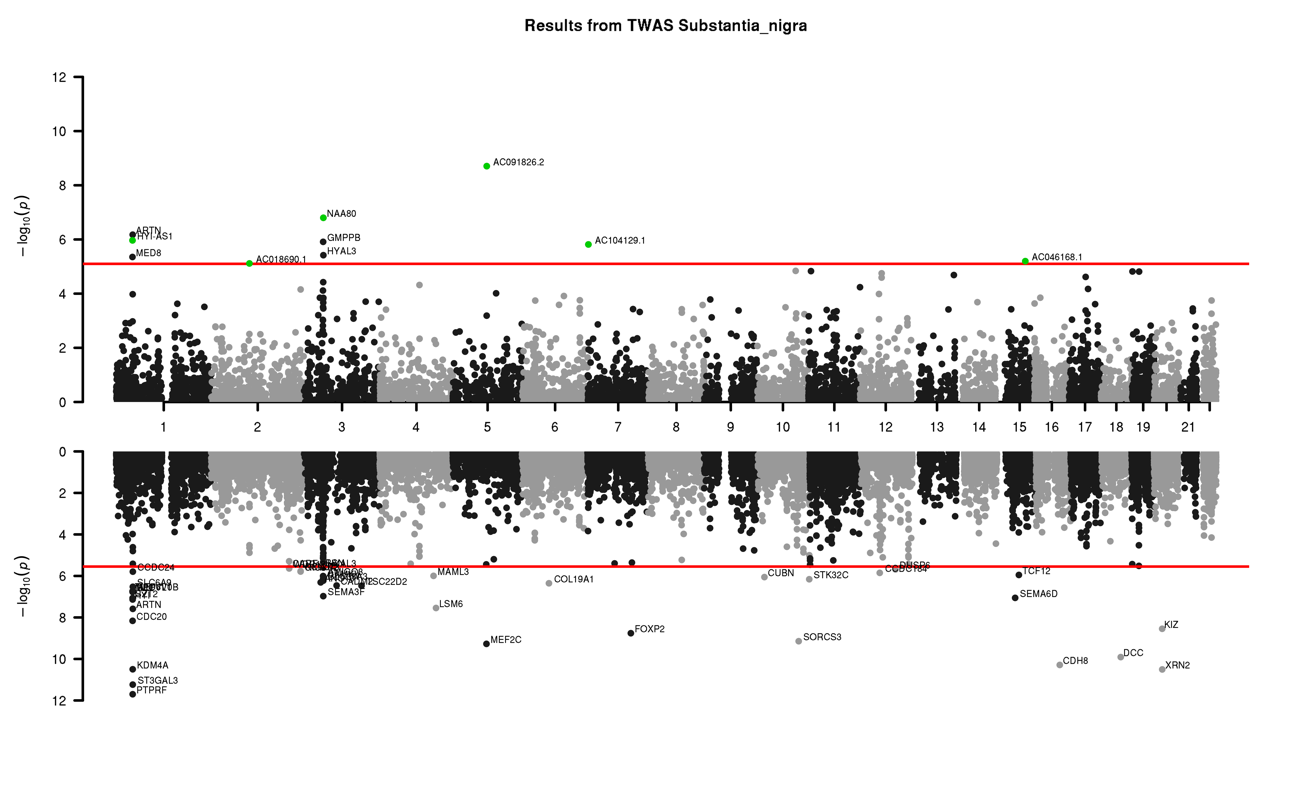


Whole blood


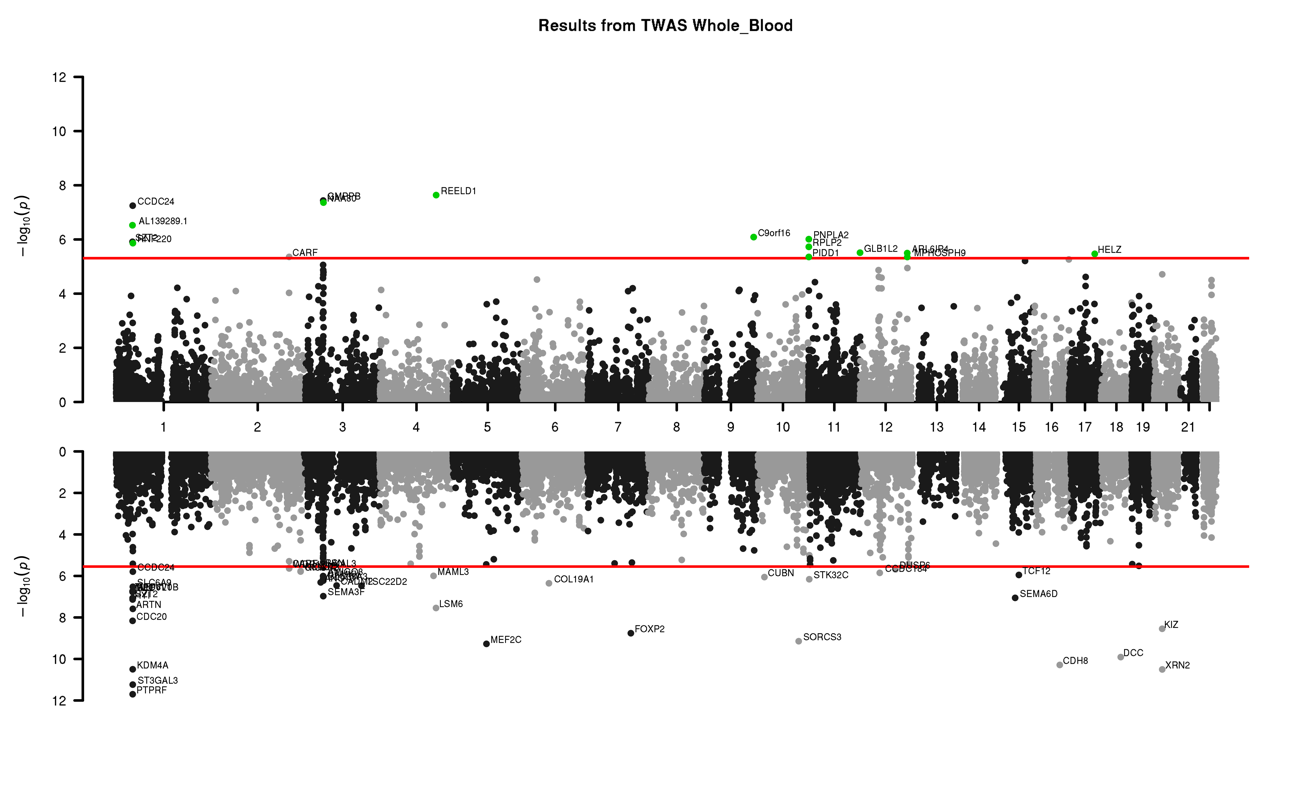


**Supplementary Figure S2. Miami plots representing the TWAS results in multiple brain tissues and whole blood.** In the upper side and results of the original gene-based GWAS on ADHD by Demontis et al. 2022 in the lower side. Highlighted in green new hits. Red lines indicate the significance threshold.

*pseudo-*R^2^

P-value threshold

*pseudo-*R^2^

**Supplementary Figure S3. Barplots showing the proportion of variance (pseudo-R2) of ADHD explained by the TRSs constructed at different TWAS P-value thresholds in multiple tissues.** Results from 13 brain tissues are shown in blue and from whole blood in orange). P-values resulting from the association between each TRS and the ADHD status is shown above each bar.

**Supplementary Figure S4. Density plots from TRSs associated with ADHD that overcome multiple comparison corrections**. Results using the best TWAS p-value threshold are shown. In red TRS distribution in individuals with ADHD and in blue TRS distribution in controls. The dashed lines indicate the mean values associated with each group.

**Supplementary Figure S5. Comparison of TRSs effect sizes before and after sensitivity analysis.** Beta estimate and 95% confidence interval from significant TRSs considering all genes (in red) and only the most significant gene per region (in black).

**Supplementary Figure S6**. **Heatmap representing correlation between TRSs and PRS.** The Spearman correlation coefficient was highlighted with a red star when the correlation was statistically significant. Represented only those TRSs that passed multiple comparison corrections and sensitivity analysis.
